# Addressing human factors in the recognition and management of local anaesthetic systemic toxicity

**DOI:** 10.1177/17504589241264403

**Published:** 2024-07-26

**Authors:** Niamh EB Curtain, Debora Gugelmin-Almeida

**Affiliations:** Faculty of Health & Social Sciences, Bournemouth University, Bournemouth, UK

**Keywords:** Local anaesthetic systemic toxicity, Human factors

## Abstract

In the perioperative environment, local anaesthetics are commonly administered to patients to provide analgesia and anaesthesia for a large range of surgical procedures. Although rare, their use can result in systemic toxicity, which is a life-threatening complication, underscoring the importance of early recognition and prompt management to mitigate patient risks. This article evaluates the impact of human factors and other aspects such as insufficient monitoring, errors in drug administration and poor adherence to safety protocols on the development and management of local anaesthetic systemic toxicity and provides practical considerations to minimise its occurrence.

## Introduction

Despite advancements in the prevention, recognition and management of local anaesthetic systemic toxicity (LAST), this adverse event remains a fatal complication of local anaesthetic (LA) administration (Neal et al 2018). LAs are commonly administered to patients undergoing surgical procedures (Fencl 2015) as they yield many benefits such as being readily available, reduced risk of polypharmacy side effects, rapid onset and cost effectiveness (Gmyrek & Dahdah 2019). Similarly, with minor procedures gradually moving from operating theatres to outpatient settings or other areas of the hospital (eg: ward, labour suites, interventional radiology), the administration of LAs in these satellite areas has also increased (Macfarlane et al 2021). Therefore, it is crucial that health care practitioners can promptly recognise LAST and are aware of current recommendations for its management.

Human factors serve as major contributors to errors in health care (Sameera et al 2021) and can play a significant role in the occurrence and management of LAST, impacting patient safety. Human factors are the various aspects that influence job performance and how people interact with one another and with technology (Brennan & Oeppen 2022). Non-technical skills form an important part of human factors mitigation strategies, encompassing skills such as situation awareness, decision-making, task management and team working (Jones et al 2018; Kelly et al 2023). By incorporating human factors into the design of systems and processes, preventable errors such as LAST can be reduced, enhancing patient safety.

## Recognition

LAST can occur when an excessive amount of the anaesthetic agent enters the systemic circulation, leading to adverse effects. Prompt recognition allows for swift intervention and mitigates potential complications. Key signs and symptoms include:

•Central nervous system (CNS) effects: agitation, restlessness, confusion, dizziness, tremors, seizures, loss of consciousness, CNS depression and coma.
•Cardiovascular effects: initial hypertension and tachycardia followed by hypotension, bradycardia, arrhythmias and cardiovascular collapse.
•Neuromuscular effects: muscle twitching and, in severe cases, muscle weakness.
•Visual and auditory disturbances: blurred vision, visual disturbances and tinnitus (Ferguson et al 2019).

Patients usually experience an excitatory phase followed by CNS depression (Brown et al 2021). It should be noted that excitatory symptoms can be challenging to recognise in the anaesthetic setting as they can be concealed by sedation or general anaesthesia (Ferguson et al 2019), differently from awake patients, who are commonly cared for in the satellite areas or outpatient environments (Gitman & Barrington 2018).

It has been recognised that LAST can occur despite strict compliance to safe practice (Christie et al 2015), which includes adherence to dosage calculation, slow administration of the LA, aspiration before injection and minimising the use of highly vascular injection sites (Kouba et al 2016, Park & Sharon 2017). This can be explained by the potential impact of human factors in the occurrence of LAST. Calculation and administration errors, inadequate monitoring, communication breakdown, fatigue or excessive workload impacting concentration and safety culture that influences the likelihood of adherence to safe practices are examples of how human factors may impact the occurrence of LAST (Jones et al 2018, Kelly et al 2023).

It is crucial for health care providers to be vigilant during procedures involving LA administration and to monitor patients closely. Timely recognition involves a combination of clinical observation, patient feedback and continuous monitoring of vital signs (Aydin 2022). In addition, ensuring that working practices are well designed and account for staffing levels, supervision arrangements and systems for identifying patient deterioration enhances patient safety during such procedures (Kelly et al 2023, Shorrock & Williams 2017).

## Management

Once LAST has been determined, prompt treatment is essential to improve safety and survival. Guidelines published by the Association of Anaesthetists (AoA) in 2023 outlined the recommended management of LAST, providing a standardised approach that accounts for potential errors and encourages teamwork and communication.

In the event of suspected LAST, immediate actions should be taken:

- Discontinue administration of the LA agent promptly.
- Notify the team and seek assistance.
- Ensure patent airway, adequate oxygenation and ventilation (with 100% oxygen).
- Cardiovascular support: administer intravenous fluids to address hypotension. In severe cases, vasopressors may be required. Lipid emulsion therapy has been reported to be a safe and beneficial treatment for LAST despite its mechanism of action remaining unclear (Christie et al 2015). It works by reducing the concentration of LA in the blood, creating a bulk lipid phase in blood plasma that reduces toxicity by scavenging LA from affected organs such as the heart and brain to large reservoir organs such as the liver or skeletal muscle (Waldinger et al 2019).
- Initiate advanced life support measures if there is cardiovascular collapse.
- Seizure may be a clinical occurrence in LAST and should be treated as soon as possible. If seizures occur, consider administering benzodiazepines (eg: midazolam).
- Continue close monitoring of vital signs, electrocardiogram (ECG) and oxygen saturation (AoA 2023).

In the management of LAST, practitioners need to make quick and accurate decisions for a prompt management. In high-stress scenarios, human factors significantly influence decision-making, posing implications for patient outcomes. Distractions, emotional responses such as fear and anxiety, communication challenges and collaboration issues can contribute to cognitive errors, potentially hindering practitioners’ ability to promptly recognise and address LAST symptoms (Aydin 2022). In addition, insufficient training, individual decision biases and poor adherence to safety practices may compromise decision-making and influence practitioners’ choices during critical moments, impacting the timeliness of interventions (Kelly et al 2023).

Addressing these implications involves comprehensive and multifactorial strategies, including ongoing education, regular simulation training, effective communication and a supportive organisational culture. There is a growing recognition that training should encompass human factors, non-technical skills and systems thinking (National Safety Standards for Invasive Procedures (NatSSIPs) 2023) as they play a crucial role in fostering safe and effective performance, thereby mitigating errors stemming from human factors. This has been observed in anaesthetic practice since the introduction of the ‘anaesthesia crisis resource management framework’ in 1990 (Radhakrishnan et al 2022) and it is growing in popularity within health care practice (Jones et al 2018). By mitigating the influence of human factors, practitioners can enhance decision-making, prevent errors, promote awareness and potentially improve patient outcomes in LAST scenarios (Edwards et al 2018, Sameera et al 2021).

## Prevention

Preventing LAST from occurring is fundamental to avoid patient morbidity and mortality associated with the use of LA (Davidson et al 2024). It is recognised that LAST is influenced by the interaction of patient-specific factors, the peak plasma anaesthetic concentration and the properties of the LA being used, impacting its occurrence and severity (Association of periOperative Registered Nurses (AORN) 2022).

According to Macfarlane et al (2021), patient-related factors include individual sensitivity to LAs, underlying health conditions and genetic predispositions. Patients with reduced hepatic or renal function may experience slower metabolism and elimination of LAs, increasing the risk of systemic toxicity. Similarly, pre-existing cardiovascular conditions can contribute to an increased vulnerability to LAST. In addition, extremes of age, such as paediatric or elderly patients, and those who are pregnant, may have altered physiological responses to LAs, impacting the occurrence and severity of LAST (Waldinger et al 2019). Furthermore, the choice of LA agent, its properties, dosage and the route of administration may elevate the risk and complications of LAST (Macfarlane et al 2021).

Preventing the occurrence of LAST involves implementing various strategies to enhance patient safety during administration of local anaesthesia including the following:

## Monitoring

Ensuring appropriate monitoring reduces the chance of delayed recognition and treatment of LAST. Although the symptoms usually manifest immediately after injection of LA, plasma levels can peak between 30 and 90 minutes after administration (El-Boghdadly et al 2018, Macfarlane et al 2021). Therefore, practitioners must be vigilant, and the patient should be monitored for a minimum of 30 minutes after the last LA dose has been administered (Neal et al 2018).

According to the AoA (2021), the minimum standards for monitoring required during regional anaesthesia should be the same as when the patient is receiving a general anaesthetic. This includes oxygen saturation, blood pressure, ECG and temperature. It is important to note that even with adequate monitoring, practitioners must remain vigilant when LA is being administered so that physiological changes can be interpreted accordingly. However, this may be impacted by factors such as fatigue, excessive workload and stress, impairing the ability to be vigilant (Jones et al 2018). Therefore, extra measures should be put in place to ensure that practitioners are protected from those factors. This includes supporting their wellbeing by ensuring adequate breaks, providing competent supervision and adhering to relevant working time legislation (AoA 2021, Kelly et al 2023).

## Safe administration

To minimise the risk of LAST, the following prevention strategies should be implemented when administering LA:

- Using the lowest effective dose to achieve the desired effect to minimise the absorption of LAs into the bloodstream.
- Injecting the LA incrementally to allow for the monitoring of patient’s response and detecting early signs of systemic toxicity.
- Aspirating the syringe before each injection to mitigate the risk of inadvertent intravascular injection and therefore systemic absorption.
- Being aware of the additive effect of LA as, when used in combination or administered sequentially, their individual effects may be intensified.
- Using ultrasound guidance when performing peripheral nerve blocks to visualise anatomical structures and make real-time adjustment.
- Discussing the dosing parameters of the LA in the preoperative briefing (AORN 2022).

It is important to note that the success of these prevention strategies is significantly influenced by human factors. For instance, the prevention of intravascular injection success depends on the attentiveness of practitioners and adherence to the correct technique (Buetti et al 2022). Distraction, fatigue, inexperience or complacency may undermine the efficacy of these prevention techniques, impacting on patient safety (NatSSIPs 2023). Similarly, the preoperative briefing proves effective in reducing errors only when information exchange involves clear and effective communication (Garrett 2016). Effective communication depends on clarity, brevity and empathy, with room for a feedback loop (Jones et al 2018). Directed and closed-loop communication proves particularly valuable as it necessitates a clear identification of the intended recipient, with the receiver required to acknowledge and report their interpretation of the information received, and the sender confirming the accuracy of the interpretation (Flemming & Carpini 2019). Fostering an environment of open information exchange becomes a strong strategy for patient safety, empowering all team members to voice their thoughts and concerns, potentially enhancing delivery of care (Etchegaray et al 2020).

## Incorporation of new technologies

The tragic case of a patient who died after giving birth, when an epidural infusion of bupivacaine was mistakenly connected to her intravenous line, highlights the issue around drugs administration, storage and handling of medications in line with standard operating procedures (Christie et al 2015, Sud & Szawarski 2018). The ‘administration of medication by the wrong route’ is considered a Never Event, which is a significant and avoidable incident that should not happen if health care providers have followed established national safety guidelines or recommendations (NHS Improvement 2018). To help mitigate the risks associated with medication administration (eg: incorrect route), new technologies have been developed.

Historically, the standard small-bore connector for medical devices has been the Luer lock, used in different procedures and devices. Due to its universal part, it allowed the wrong route administration of fluids and gasses into patients, sometimes with harmful or fatal results (Cannons & Shaw 2021). To minimise this risk, NRFit^™^ connector has been developed for injecting into the neuraxial space and for peripheral nerve blocks (Nair & Diwan 2021). These connectors are smaller in diameter and longer in length when compared to the standard Luer lock, reducing the occurrence of misconnections. The implementation of NRFit^™^ is a global initiative, with hospitals across the world making the change to promote patient safety in regional anaesthesia (NHS England 2024).

Other initiatives include smart infusion pumps with dose-error reduction systems to provide precise drug delivery (AAMI 2015) and digital health platforms incorporating patient monitoring and data analytics, enabling for continuous surveillance and allowing for early detection of adverse events and prompt intervention (Yeung et al 2023).

Despite the risk of medication errors being potentially minimised by the development of new technologies, its effectiveness is dependent on consistent implementation and adherence by health care professionals. The willingness to accept and embrace changes in medication practices can be influenced by resistance to change and ingrained habits (Cheraghi et al 2023). Consistent and accurate implementation requires attention to detail and adherence to established protocols, all of which can be influenced by human factors such as workload, stress and competing priorities.

## Engaging in safety initiatives

In an attempt to avoid errors in health care practice, many safety initiatives have also been created and implemented. A relevant example includes ‘Stop Before You Block’, which emphasises the importance of practitioners pausing and thoroughly verifying critical elements before administering a nerve block (SALG 2021). While the ‘Stop Before You Block’ initiative primarily focuses on preventing wrong-side blocks and ensuring the accuracy of nerve block administration, it indirectly contributes to patient safety, including the avoidance of LAST. By emphasising the importance of pausing, verifying and confirming critical details before administering anaesthesia, this practice helps in reducing the risk of procedural errors, including those that could lead to LAST as it aligns with the broader goal of promoting a thorough and systematic approach to patient safety (NatSSIPs 2023).

However, to ensure effective engagement with safety initiatives, human factors that may hinder their benefits should be considered. Distractions divert practitioners’ attention and can lead to oversights; communication breakdown among team members can undermine the effectiveness of safety protocols; time constraints may add pressure and compel practitioners to expedite procedures; complacency due to familiarity with routine procedures can result in a relaxed attitude towards safety checks, increasing the likelihood of oversights (Cheraghi et al 2023, Kelly et al 2023). These examples of individual cognitive biases can introduce errors and compromise the effectiveness of safety measures.

## Education and training

Education and training can help practitioners develop the necessary skills and knowledge to improve clinical practice and prevent human error (CIEHF 2018, HEE 2016). The development of comprehensive and ongoing education and training programmes that focus on the prevention, recognition and management of LAST can ensure that patients are treated promptly. Bevil et al (2020) conducted a study to assess perioperative nurses’ retention of knowledge regarding LAST using high-fidelity simulation along with a brief didactic session. The findings demonstrate that the simulation increased participants’ knowledge base, enhanced communication and improved teamwork, which may increase the likelihood of early recognition of LAST and improve the chances of a positive outcome.

In addition, human factors training is also important so that health care professionals can enhance their understanding of how cognitive, social and organisational factors influence performance and ultimately patient safety. Research carried out by Buljac-Samardzic et al (2020) highlights the significance of such training within health care, aiming to recognise the value of non-technical skills such as communication, situational awareness, decision-making and teamwork, in the prevention of errors. This type of training empowers health care teams to cultivate a culture of safety, improve communication and teamwork and mitigate the risks associated with human factors (Jones et al 2018).

## Organisational culture

By promoting the use of checklists, safety initiatives and thorough documentation to enforce safety measures, hospitals and other health care settings, such as outpatient clinics, can cultivate an organisational culture that prioritises continuous quality improvement (NatSSIPs 2023). This includes fostering a practice where staff are encouraged to report incidents and near-misses without fear of reprisal, allowing for a transparent and open approach to learning from errors (van Baarle et al 2022). By actively involving staff in the analysis of adverse events and near-misses, organisations can empower employees to take ownership of safety initiatives and contribute to the development of effective solutions. Constructive collaboration and communication within interdisciplinary teams are also vital components of a strong safety culture (Buljac-Samardzic et al 2020), as they facilitate the sharing of information, ideas and concerns among team members. This collaborative approach fosters a sense of collective responsibility for patient safety and encourages proactive problem-solving to address potential risks or issues. Ultimately, by embedding a culture of safety into the organisation’s values and principles, health care practitioners can create an environment where patient safety is paramount, and continuous improvement is the standard practice.

## Conclusion

LAST is a rare yet significant complication of regional anaesthesia, often influenced by human factors. Addressing the impacts of human factors and implementing a multifaceted approach involving effective monitoring, appropriate administration, implementation of new technologies, adherence to safety protocols and engagement in relevant training can potentially improve the standard of care delivered by health care professionals and enhance patient outcomes.
